# Serum Perilipin 2 (PLIN2) Predicts Multiple Organ Dysfunction in Critically Ill Patients

**DOI:** 10.3390/biomedicines9091210

**Published:** 2021-09-13

**Authors:** Berkan Kurt, Lukas Buendgens, Theresa H. Wirtz, Sven H. Loosen, Maximilian Schulze-Hagen, Daniel Truhn, Jonathan F. Brozat, Samira Abu Jhaisha, Philipp Hohlstein, Ger Koek, Ralf Weiskirchen, Christian Trautwein, Frank Tacke, Karim Hamesch, Alexander Koch

**Affiliations:** 1 Department of Medicine III, University Hospital RWTH Aachen, Pauwelsstraße 30, 52074 Aachen, Germany; bkurt@ukaachen.de (B.K.); lbuendgens@ukaachen.de (L.B.); thwirtz@ukaachen.de (T.H.W.); jbrozat@ukaachen.de (J.F.B.); sabujhaisha@ukaachen.de (S.A.J.); phohlstein@ukaachen.de (P.H.); ctrautwein@ukaachen.de (C.T.); khamesch@ukaachen.de (K.H.); 2 Clinic for Gastroenterology, Hepatology and Infectious Diseases, University Hospital Düsseldorf, Moorenstaße 5, 40225 Düsseldorf, Germany; sven.loosen@med.uni-duesseldorf.de; 3 Department of Diagnostic and Interventional Radiology, University Hospital of RWTH Aachen, Pauwelsstraße 30, 52074 Aachen, Germany; mschulze@ukaachen.de (M.S.-H.); dtruhn@ukaachen.de (D.T.); 4 Department of Internal Medicine, Division of Gastroenterology and Hepatology, Maastricht University Medical Centre (MUMC), 6229 HX Maastricht, The Netherlands; gh.koek@mumc.nl; 5 Institute of Molecular Pathobiochemistry, Experimental Gene Therapy and Clinical Chemistry (IFMPEGKC), University Hospital of RWTH Aachen, Pauwelsstraße 30, 52074 Aachen, Germany; rweiskirchen@ukaachen.de; 6 Department of Hepatology and Gastroenterology, Charité-Universitätsmedizin Berlin, 10117 Berlin, Germany; frank.tacke@charite.de

**Keywords:** sepsis, critical illness, intensive care unit, biomarker, SOFA, prognosis, outcome, organ failure

## Abstract

Perilipin 2 (PLIN2) is a lipid droplet protein with various metabolic functions. However, studies investigating PLIN2 in the context of inflammation, especially in systemic and acute inflammation, are lacking. Hence, we assessed the relevance of serum PLIN2 in critically ill patients. We measured serum PLIN2 serum in 259 critically ill patients (166 with sepsis) upon admission to a medical intensive care unit (ICU) compared to 12 healthy controls. A subset of 36 patients underwent computed tomography to quantify body composition. Compared to controls, serum PLIN2 concentrations were elevated in critically ill patients at ICU admission. Interestingly, PLIN2 independently indicated multiple organ dysfunction (MOD), defined as a SOFA score > 9 points, at ICU admission, and was also able to independently predict MOD after 48 h. Moreover, serum PLIN2 levels were associated with severe respiratory failure potentially reflecting a moribund state. However, PLIN2 was neither a predictor of ICU mortality nor did it reflect metabolic dysregulation. Conclusively, the first study assessing serum PLIN2 in critical illness proved that it may assist in risk stratification because it is capable of independently indicating MOD at admission and predicting MOD 48 h after PLIN2 measurement. Further evaluation regarding the underlying mechanisms is warranted.

## 1. Introduction

Perilipin 2 (PLIN2), also referred to as Adipose differentiation-related protein (ADRP) or Adipophilin, belongs to the PAT family of lipid droplet proteins, which is named after the first three proteins that were discovered in this group (Perilipin (PLIN), ADRP/PLIN2 and TIP47 (Tail-interacting protein of 47 kDa)/PLIN3). PLIN2 is not only expressed in adipocytes, but also ubiquitously in non-adipose tissue (e.g., cardiomyocytes and skeletal muscle cells, hepatocytes, intestinal cells and endothelial cells) [1,2,3,4,5,6].

Although PLIN2′s main function is the regulation of lipid metabolism as the regulation of intracellular lipid storage and lipolysis [5,7,8,9], recent evidence uncovered various additional functions. A growing body of studies demonstrates the complex regulation and pathophysiological connections of PLIN2 in lipid metabolism and beyond [10,11]. For instance, experimental and clinical data suggest that PLIN2 is involved in the pathophysiology of insulin resistance and type 2 diabetes mellitus [12,13,14], dyslipidemia [5,7,15,16] and fatty liver disease [7,14,17,18,19,20,21,22]. Additionally, there has been evidence connecting PLIN2 to the development of age-related vascular disease, such as atherosclerosis [15,23,24,25,26,27]. Moreover, PLIN2 is also important in regulating lipid accumulation in cardiomyocytes [28]. In addition to metabolic and cardiovascular associations, PLIN2 has been described as a potential tumor marker in prevalent malignancies such as colorectal or lung carcinoma [29,30]. Furthermore, recent data suggested a possible connection between PLIN2, muscle weakness and age-related sarcopenia via a mechanism of excessive intramuscular triglyceride storage [31,32,33,34].

Disorders in lipid metabolism contribute to chronic inflammatory processes leading to metabolic diseases [35] and, in turn, metabolic comorbidities were shown to contribute to mortality and morbidity in critically ill patients on the intensive care unit (ICU) [36]. Hence, a biomarker reflecting these pathogenic processes may provide better prediction of critical illness outcomes. Given the described associations with multiple systemic, age-related and metabolic diseases, it is tempting to speculate that PLIN2 may reflect metabolic dysregulation and a moribund state in critical illness. However, no assessment currently exists of PLIN2 in critically ill patients and sepsis. This prompted us to analyze the usefulness of serum PLIN2 in a large cohort of well-characterized critically ill patients admitted to a medical ICU.

## 2. Materials and Methods

### 2.1. Study Design and Patient Characteristics

The study was constructed as a prospective, explorative, observational cohort study aiming to evaluate the role of Perilipin 2 (PLIN2) serum levels in critically ill patients in the intensive care unit. In the period from 2006 to 2011, all patients were enrolled at admission to a medical intensive care unit of the University Hospital RWTH Aachen. Study-specific exclusion criteria were (i) age below 18 years, (ii) length of stay less than 24 h, (iii) admission due to post-operative or post-interventional observation, (iv) pregnancy, (v) breastfeeding, and (vi) missing informed consent. An over-the-phone follow up with the patient or their relatives was conducted to collect information about outcomes until 2011. Serum PLIN2 was analyzed in 259 patients with available follow-up data. The reference group consisted of 12 healthy blood donors without a known acute or chronic illness, without any chronic medication, with negative serological testing for HIV and viral hepatitis, and with negative inflammatory serum markers and unremarkable biochemical workup. Diagnosis of sepsis was determined retrospectively by use of “The Third International Consensus Definitions for Sepsis and Septic Shock (Sepsis-3)” [37] and the treatment was conducted according to current guidelines at that time. Blood biochemistry markers, clinical data (e.g., on ventilation and medication) and composite scores (e.g., “Sequential Organ Failure Assessment Score” (SOFA) and “Acute Physiology And Chronic Health Evaluation II” (APACHE-II)) [37,38] were used to evaluate the presence of organ dysfunction. Standard biochemistry blood markers were taken for clinical routine measurements and analyzed in the central laboratory at University Hospital RWTH Aachen with the use of Sysmex XN9000 (Sysmex GmbH, Norderstedt, Germany) and Cobas 8000 c701 (Hoffmann-La Roche AG, Basel, Switzerland). Ethical approval was provided by the institutional review board of the University Hospital RWTH Aachen University (Aachen, Germany; reference number: EK 150/06; date of approval: 2 November 2006). Written informed consent was obtained by the patients or their legal representatives. The study was executed in accordance with the ethical guidelines of the Declaration of Helsinki (Hong Kong Amendment) and Good Clinical Practice (European guidelines).

### 2.2. PLIN2 Measurements

Blood samples were collected at admission to the ICU after written informed consent was obtained. After centrifugation (at 4 °C for 10 min) and aliquotation, the serum samples were immediately transferred to a −80 °C freezer. For the measurements of serum PLIN2, a commercial quantitative sandwich enzyme immunoassay (ELISA) was used according to the manufacturer’s instructions (Uscn Life Science. Inc., Wuhan, China; No: E91350Hu). The minimum detectable dose of PLIN2 was less than 0.0053 µg/dL. The determined sensitivity of this assay (i.e., the lower limit of detection) as given by the manufacturer was determined by adding three standard deviations to the mean optical density value of twenty zero standard replicates and calculating the corresponding concentration. The instructions of the manufacturer state high sensitivity and specificity for detection of human PLIN2 with no further quantification. No significant cross-reactivity or interference between human PLIN2 and analogues was observed. The successor test (No: SEB350Hu) has comparable specifications: the minimum detectable dose of PLIN2 is less than 0.0055 µg/dL. The determined sensitivity of this assay (i.e., the lower limit of detection) as given by the manufacturer was determined by adding two standard deviations to the mean optical density value of twenty zero standard replicates and calculating the corresponding concentration. The intra-assay and inter-assay precisions are lower than 10% and 12%, respectively. No significant cross-reactivity or interference between PLIN2 and analogue are observed in this double-antibody sandwich ELISA [39]. Measurements were performed by experienced laboratory personnel that was fully blinded to any clinical or other laboratory data of the patients or controls.

### 2.3. Assessment of Computed Tomography Scan Body Composition Markers

Data from patients who received a computed tomography (CT) scan at admission to our ICU were included in our analysis. CTs were performed on either a 40-row spiral CT scanner (Somatom Definition AS 40, Siemens Medical Systems, Erlangen, Germany) or 128-row spiral CT scanners (Somatom Definition Flash or Somatom Definition AS, Siemens Medical Systems, Germany). The scans were acquired in the craniocaudal direction during a single breath-hold with a tube voltage of 120 kV and automated tube current modulation. Reconstructed slice thickness was 5 mm and only venous-phase scans were used for body composition calculations. According to the literature, there is a strong association between single-slice measurements and total compartment volumes [40,41,42]. Hence, total visceral and subcutaneous adipose tissue (VAT, SAT), skeletal muscle area and its mean attenuation given in Hounsfield units (HU) were segmented at the center plane of the third lumbar vertebra on axial CT scans. The semi-automatic segmentation tool “3D slicer”, an open-source software application for medical image computing, was used to determine the given body composition markers [43]. The skeletal muscle index was calculated for standardization purposes via dividing the skeletal muscle area by the corresponding body height.

### 2.4. Statistical Analysis

All categorical variables were described as absolute (n) and relative (%) frequencies and the corresponding contingency tables were analyzed with chi-square tests or Fisher’s exact test if *n* ≤ 5. Continuous variables were displayed as stated (mainly median and range) and were analyzed by the Mann–Whitney U test. The Kruskal–Wallis *H* test was used to analyze differences for multiple comparisons. Correlation analyses (e.g., between clinical variables and blood biomarkers) were assessed using Spearman’s Rho correlation coefficient (*r*). Graphical illustration of data was performed via box plots including median, quartiles and whiskers to indicate the range of values. The median PLIN2 serum level was used as a cut-off for subsequent analyses. Time-to-event variables were displayed using Kaplan–Meier curves and differences in survival were tested using the log-rank test. To analyze the independent value of PLIN2 as a prognostic biomarker, univariable and multivariable analyses of median PLIN2 serum level for outcomes such as sepsis and multiple organ dysfunction (MOD) were conducted using logistic regression analyses to calculate odds ratios (*OR*). Distributions among groups were assessed by univariable and forward-stepwise multiple logistic regression analyses to calculate *OR*. Multivariable logistic regression analyses were performed to test for independent associations. *OR*s were presented with their corresponding 95% confidence intervals (CI) given in brackets. *p*-values < 0.05 were considered statistically significant whereas *p*-values > 0.10 were given as “n.s.” (not significant) to offer better readability. The data were analyzed and visualized using SPSS Statistics version 27 (IBM; Armonk, NY, USA).

## 3. Results

### 3.1. PLIN2 Serum Levels Were Significantly Elevated in Critically Ill Patients

**Figure 1:**
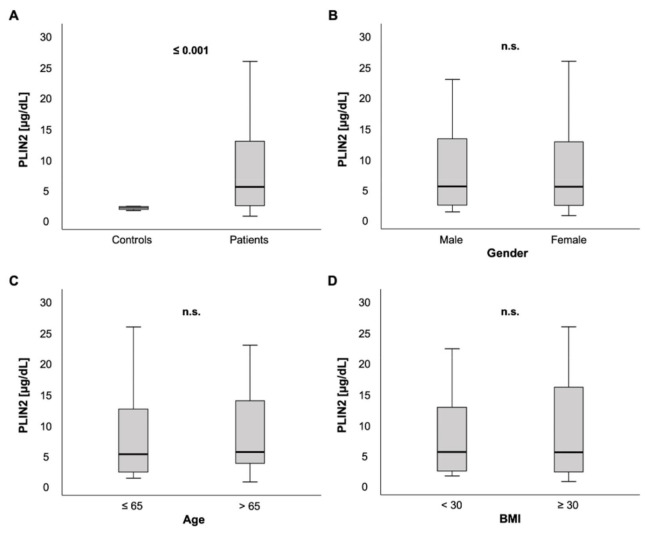
PLIN2 serum concentrations in critically ill patients at intensive care unit (ICU) admission. (**A**) Critically ill patients on the ICU had elevated PLIN2 serum levels compared to healthy controls. (**B**–**D**) No difference of PLIN2 serum concentrations was seen between male and female ICU patients (**B**), ICU patients ≤ 65 vs. >65 years (**C**) or ICU patients with a body mass index (BMI) < 30 vs. ≥30 kg/m^2^ (**D**).

We measured PLIN2 serum concentrations in 259 patients on ICU admission. In comparison to a healthy control group consisting of 12 blood donors free from any chronic comorbidities or laboratory abnormalities, PLIN2 serum levels were markedly elevated in ICU patients (5.23 (0.48–59.5) µg/dL vs. 1.83 (1.36–2.07) µg/dL, *p* < 0.001; Figure 1A).

Among ICU patients, no significant difference in PLIN2 serum concentrations was observed between male and female patients (5.27 (0.11–59.5) µg/dL vs. 5.21 (0.48–48.3) µg/dL, *p* = 0.562; Figure 1B). PLIN2 serum levels neither showed a significant difference between patients that were ≤65 years old versus patients > 65 years (5.02 (0.11–59.5) µg/dL vs. 5.39 (0.48–48.3) µg/dL, *p* = 0.535; Figure 1C) nor did it correlate directly with age (*r =* 0.063, *p* = 0.305; Table 1).

### 3.2. Associations of PLIN2 Serum Concentrations with Clinical Data and Blood-Based Parameters

Because PLIN2 is described to be essential in the formation of cytoplasmic lipid droplets [3,15,44], we hypothesized that PLIN2 concentrations may be associated with obesity and established lipid markers. However, no significant difference was observed between ICU patients with a BMI ≥ 30 kg/m^2^, defining obesity, versus < 30 kg/m^2^ (5.3 (1.4–59.5) µg/dL vs. 5.27 (0.48–57.1) µg/dL, *p* = 0.570; Figure 1D). In line with this, there was no correlation between serum PLIN2 and BMI (*r =* −0.099, *p* = 0.129; Table 1) and between different BMI classes (<18.5, 18.5–24.9, 25–29.9, ≥30 kg/m^2^, *p* = 0.678, data not shown). Furthermore, we compared serum PLIN2 with various parameters of lipid metabolism. Unexpectedly, we did not observe any associations between PLIN2 levels and total cholesterol (*r =* −0.011, *p* = 0.868), high density lipoprotein (HDL) cholesterol (*r =* 0.062, *p* = 0.533) or low-density lipoprotein (LDL) cholesterol (*r =* 0.101, *p* = 0.309).

**Figure 2:**
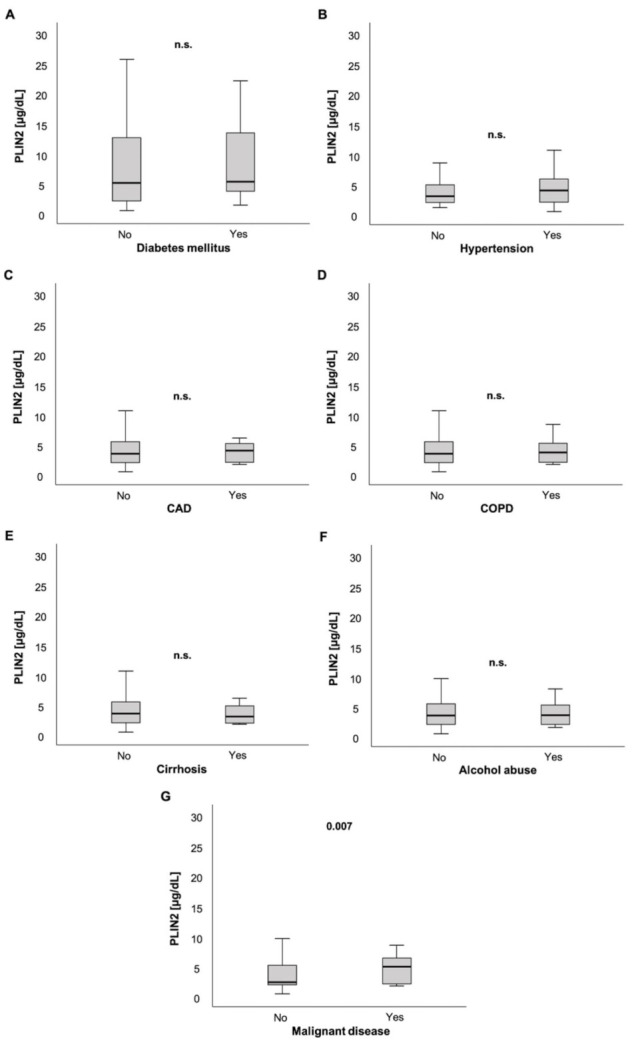
PLIN2 serum levels and preexisting chronic comorbidities. PLIN2 levels were not significantly different in ICU patients with preexisting diabetes mellitus, arterial hypertension, coronary artery disease, chronic obstructive pulmonary disease, liver cirrhosis and chronic alcohol abuse compared to their counterparts without these comorbidities (**A**–**F**). ICU patients with pre-diagnosed malignant disease had significantly elevated serum PLIN2 levels (**G**).

Next, we analyzed parameters of glucose metabolism. Serum PLIN2 levels were not elevated in patients with diabetes mellitus (5.13 (0.48–59.5) µg/dL vs. 5.32 (1.4–22.0) µg/dL, *p* = 0.287; Figure 2A). Moreover, we did not observe correlations between PLIN2 serum levels and glycated hemoglobin (HbA1c, *r =* −0.090, *p* = 0.355; Table 1) or homeostasis model assessment-insulin resistance (HOMA-IR, *r =* −0.173, *p* = 0.077; Table 1).

While ICU patients with arterial hypertension (AH) showed a trend of having higher serum PLIN2 levels compared to their counterparts without AH (3.1 (1.1–39.3) µg/dL vs. 4 (4.8–48.3) µg/dL, *p* = 0.072; Figure 2B), patients with coronary artery disease (CAD) showed no significant difference in PLIN2 concentrations compared to patients without CAD (3.5 (0.48–48.3) µg/dL vs. 4.02 (1.69–21.3) µg/dL, *p* = 0.567; Figure 2C).

Furthermore, PLIN2 serum levels did not differ in patients with and without chronic obstructive pulmonary disease (COPD) (3.5 (0.48–48.3) µg/dL vs. 3.74 (1.68–21.3) µg/dL, *p* = 0.974; Figure 2D).

ICU patients with or without liver cirrhosis did not have different serum PLIN2 levels (3.56 (0.48–48.3) µg/dL vs. 3.11 (1.78–6.07) µg/dL, *p* = 0.595; Figure 2E). Similarly, a history of alcohol abuse did not show altered PLIN2 levels in ICU patients (3.52 (0.48–48.3) µg/dL vs. 3.61 (1.53–40) µg/dL, *p* = 0.860; Figure 2F).

Surprisingly, we did not observe any significant correlations between traditional markers of inflammation and PLIN2 concentrations (WBC: *r =* −0.109, *p* = 0.080; C-reactive protein: *r =* 0.025, *p* = 0.688; Procalcitonin: *r =* −0.023, *p* = 0.751; Interleukin 6: *r =* 0.050, *p* = 0.486; TNF-α: *r =* −0.165, *p* = 0.157).

Serum PLIN2 had a strong and negative correlation with lipase (*r =* −0.509, *p* < 0.001). Corresponding to that, compared to ICU patients without acute pancreatitis, PLIN2 concentrations were reduced in patients admitted due to acute pancreatitis (5.31 (0.48–59.5) µg/dL vs. 2.07 (1.59–32.4) µg/dL, *p* = 0.042; Figure S1).

Interestingly, in contrast to ICU patients without active malignancy, ICU patients with active malignant disease had higher serum PLIN2 concentrations (2.45 (0.48–40) µg/dL vs. 5.02 (1.78–48.3) µg/dL, *p* = 0.007; Figure 2G).

Finally, we compared the serum concentrations of PLIN2 with experimental biomarkers (Table S1). Adipokines such as Adiponectin emerged as a new and promising group of inflammatory biomarkers in intensive care medicine [45,46]. PLIN2 levels showed an inverse correlation with Adiponectin (*r =* −0.273, *p* = 0.007) and a correlation with Myostatin (*r =* 0.160, *p* = 0.010). Furthermore, we detected a correlation of PLIN2 with Asymmetric dimethylarginine (ADMA) (*r =* −0.197, *p* = 0.002) and Symmetric dimethylarginine (SDMA) (*r =*−0.154, *p* = 0.017), two endogenous nitric oxide modulators.

### 3.3. PLIN2 Levels Were Associated with Organ Failure and Disease Severity

Because sepsis was a main cause for admission to our medical ICU (Table S2), we assessed whether PLIN2 levels were associated with sepsis occurrence. ICU patients with sepsis had comparable baseline characteristics and comorbidities as their counterparts without sepsis (Table 2). Septic ICU patients had a higher demand for mechanic ventilation, vasopressor therapy and renal replacement (Table 2). In terms of laboratory parameters, septic patients had higher levels of inflammatory markers (e.g., white blood cell count, C-reactive protein and procalcitonin), elevation of kidney function parameters (e.g., creatinine), higher norepinephrine demand, and higher SOFA and APACHE-II scores on the day of ICU admission (Table 2). Moreover, septic ICU patients had a longer ICU stay, higher ICU mortality and higher overall mortality (Table 2).

**Figure 3:**
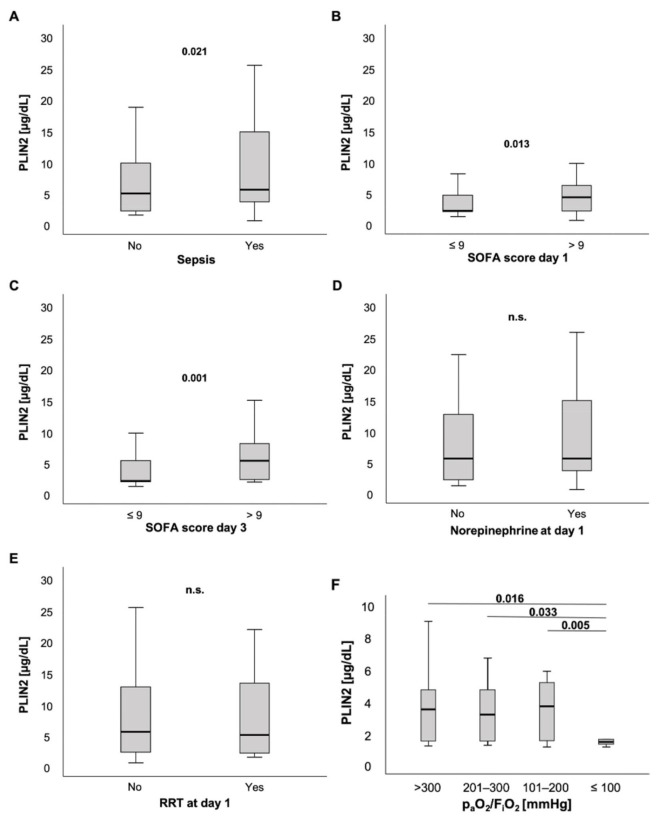
PLIN2 levels in different subsets of ICU patients. (**A**) PLIN2 serum concentrations were elevated in ICU patients with sepsis. (**B**) ICU patients with a SOFA score > 9 points had higher serum PLIN2 levels. (**C**) Serum PLIN2 on the day of ICU admission was higher in ICU patients with a SOFA score > 9 points at ICU day 3. (**D**,**E**) Neither patients with norepinephrine (**D**) nor renal replacement therapy demand showed differences in PLIN2 levels (**E**). (**F**) Serum PLIN2 levels in the different PaO_2_/FiO_2_ (Horovitz index) subgroups are shown.

PLIN2 serum levels were slightly elevated in ICU patients with sepsis compared to ICU patients without sepsis (5.47 (0.48–595) µg/dL vs. 4.86 (1.4–32.4) µg/dL, *p* = 0.021; Figure 3A). Moreover, patients with high sepsis disease severity and multiple organ dysfunction (MOD), defined as a SOFA score > 9 points on the day of ICU admission, had markedly higher PLIN2 concentrations at ICU admission than their counterparts with SOFA ≤9 points (4.22 (0.48–22) µg/dL vs. 2.05 (1.1–48.3) µg/dL, *p* = 0.013; Figure 3B). Of note, these findings were independent of the infectious site (Figure S2). Next, we tested whether PLIN2 was able to predict MOD at farther time points during ICU stay. Interestingly, serum PLIN2 levels on the day of ICU admission were higher in those ICU patients who had a SOFA score > 9 points on the third day of ICU stay, i.e., 48 h after the PLIN2 measurement, compared to ICU patients who had a SOFA score ≤9 points on ICU day 3 (5.2 (1.82–32.4) µg/dL vs. 2.02 (1.1–48.3) µg/dL, *p* = 0.001; Figure 3C). Corresponding to that, PLIN2 levels correlated with clinical parameters such as the length of ICU stay (*r =* 0.132, *p* = 0.034), and SOFA and APACHE-II scores at day 3 (*r =* 0.261, *p* = 0.014; *r =* 0.240, *p* = 0.020; respectively).

To understand whether a specific organ dysfunction was associated with increased PLIN2 levels, we dissected the individual components incorporated into the SOFA score.

ICU patients without or with norepinephrine demand at admission to the ICU did not show any difference in PLIN2 concentrations (5.47 (1.1–57.1) µg/dL vs. 5.47 (0.48–59.5) µg/dL, *p* = 0.467; Figure 3D). Because norepinephrine-induced lipolysis may be a source for PLIN2, we also calculated the standardized norepinephrine demand over 24 h. The norepinephrine concentration (µg/kg/min) did not correlate with serum PLIN2 concentrations (*r =* 0.066, *p* = 0.322; Table 1).

Additionally, there was no difference between patients with and without requirement of renal replacement therapy (5.47 (0.48–59.5) µg/dL vs. 4.95 (1.4–57.1) µg/dL, *p* = 0.447; Figure 3E). However, anuric patients presented with significantly decreased PLIN2 concentrations when compared to patients with urine output ≥100 mL per day (2.09 (1.4–20.5) µg/dL vs. 5.47 (0.48–59.5) µg/dL, *p* = 0.013; Figure S3A). We observed that this difference was stronger at day 3 (2.01 (1.53–10.23) µg/dL vs. 5.87 (0.48–76.8) µg/dL, *p* < 0.001; Figure S3B).

Another organ system reflected in the SOFA score, namely, coagulation (platelets), showed a weak correlation with serum PLIN2 levels (*r =* −0.135, *p* = 0.030).

Notably, among critically ill patients presenting with a PaO_2_/FiO_2_ ratio (Horovitz index) ≤ 100 mmHg, PLIN2 serum levels were decreased compared to ICU patients with a PaO_2_/FiO_2_ ratio > 100 mmHg (Horovitz index: >300 vs. ≤100: 4.02 (1.74–39.3) µg/dL vs. 2.01 (0.48–7.9) µg/dL; 201–300 vs. ≤100: 3.69 (1.78–13.5) µg/dL vs. 2.01 (0.48–7.9) µg/dL; 101–200 vs. ≤100: 4.23 (1.69–17.2) µg/dL vs. 2.01 (0.48–7.9) µg/dL; *p* = 0.016, *p* = 0.033, *p* = 0.005, respectively; Figure 3F). Further analyses revealed that demand of FiO_2_ levels during ventilation correlated with PLIN2 concentrations (*r =* −0.224, *p* = 0.026). Additionally, there was a trend towards a weak but insignificant correlation between PLIN2 levels and the Horovitz quotient (*r =* 0.202, *p* = 0.052).

We performed uni- and multivariable logistic regression analyses to evaluate whether PLIN2 acts as an independent marker for occurrence of sepsis and disease severity (i.e., multiple organ dysfunction (MOD)) (Table 3). To test for independent association of serum PLIN2, we performed various multivariable logistic regression analyses accounting for different parameters that were either associated with PLIN2 serum levels in the literature and/or are associated with sepsis [6,18,32,47,48], i.e., diabetes mellitus (DM) occurrence and age, BMI, norepinephrine demand and established laboratory markers for inflammation, such as C-reactive protein (CRP) and procalcitonin (PCT).

For indicating sepsis, PLIN2 serum levels above the median had a sensitivity of 55.4%, a specificity of 58.1%, a positive predictive value (PPV) of 70.2% and a negative predictive value (NPV) of 42.2%. Sepsis occurrence at ICU admission was weakly associated with elevated PLIN2 levels above the median concentration in the unadjusted analysis (*OR =* 1.72 (1.03–2.88), *p* = 0.038; Table 3, upper panel). Presence of sepsis at ICU admission was still associated with serum PLIN2 above the median concentration after multivariable adjustment for age and DM (*OR =* 1.71 (1.02–2.87), *p* = 0.042; Table 3, upper panel). The significance of association was lost after adjustment for CRP (*OR =* 1.75 (0.94–3.28), *p* = 0.079; Table 3, upper panel) and after adjustment for CRP and PCT (*OR =* 2.07 (0.98–4.38), *p* = 0.058; Table 3, upper panel). However, serum PLIN2 was significantly associated with sepsis occurrence at ICU admission after adjustment for norepinephrine demand (*OR =* 1.73 (1.01–2.98), *p* = 0.047; Table 3, upper panel).

Next, we analyzed whether serum PLIN2 was associated with MOD, defined as SOFA score > 9 points, at the time of ICU admission. For indicating SOFA > 9 points, serum PLIN2 above the median had a sensitivity of 43.4%, a specificity of 80.3%, a PPV of 65.7% and a NPV of 62%. In the unadjusted analysis, serum PLIN2 levels above the median concentration were clearly associated with MOD (*OR =* 3.13 (1.36−7.20); *p* = 0.007; Table 3, middle panel). MOD was still associated with serum PLIN2 concentrations above the median after adjustment for age and DM (*OR =* 2.96 (1.27–6.88), *p* = 0.012; Table 3, middle panel). PLIN2′s association with MOD at ICU admission remained significant after adjustment for CRP (*OR =* 3.07 (1.33–7.09), *p* = 0.009; Table 3, middle panel); however, this was lost after further adjustment for CRP and PCT (*OR =* 2.62 (0.67–10.24), *p* = 0.166; Table 3, middle panel). Moreover, the association between PLIN2 and MOD at ICU admission remained significant after adjustment for norepinephrine demand (*OR =* 2.72 (1.08–6.89), *p* = 0.035; Table 3, middle panel).

Finally, we evaluated whether serum PLIN2 concentrations above the median at the time of ICU admission were associated with SOFA score > 9 points, indicating MOD, on day 3 after admission (i.e., 48 h after PLIN2 measurement). Serum PLIN2 above the median had a sensitivity of 50%, a specificity of 75.5%, a PPV of 58.1% and a NPV of 69.0%. In the unadjusted analysis, there was a strong association between serum PLIN2 above the median concentration at ICU admission and SOFA > 9 points at day 3 (*OR =* 3.08 (1.25–7.60), *p* = 0.015; Table 3, lower panel). This association remained significant after multivariable adjustment for age and DM (*OR =* 2.91 (1.16–7.29), *p* = 0.023; Table 3, lower panel). Furthermore, the association between PLIN2 and MOD on day 3 remained significant after adjustment for CRP (*OR =* 2.93 (1.17–7.32), *p* = 0.021; Table 3, lower panel) and after adjustment for CRP and PCT (15.93 (2.85–88.93), *p* = 0.002; Table 3, lower panel). Moreover, the association between serum PLIN2 above median concentrations at ICU admission and SOFA score > 9 points on day 3 remained significant after adjustment for norepinephrine demand (*OR =* 2.79 (1.05–7.42), *p* = 0.040; Table 3, lower panel).

### 3.4. PLIN2 Serum Levels and Association with CT Scan Body Composition Markers

A subset of 36 patients, who underwent computed tomography scans, was used to assess body composition (Table 4). While the mean skeletal muscle attenuation given in Hounsfield units was used as a surrogate parameter for myosteatosis, the normalized skeletal muscle index at L3 (L3SMI) served to evaluate sarcopenia. In the small subset of patients with a BMI ≥ 30 kg/m^2^ (*n* = 9), we detected an association of serum PLIN2 with visceral adipose tissue in (*r =* −0.750, *p* = 0.020; Table 4). Besides that, we did not observe any significant correlations between CT scan body composition markers and PLIN2 serum concentrations.

### 3.5. Serum PLIN2 Concentrations May Predict ICU Mortality in Critically Ill Patients Older Than 65 Years

**Figure 4:**
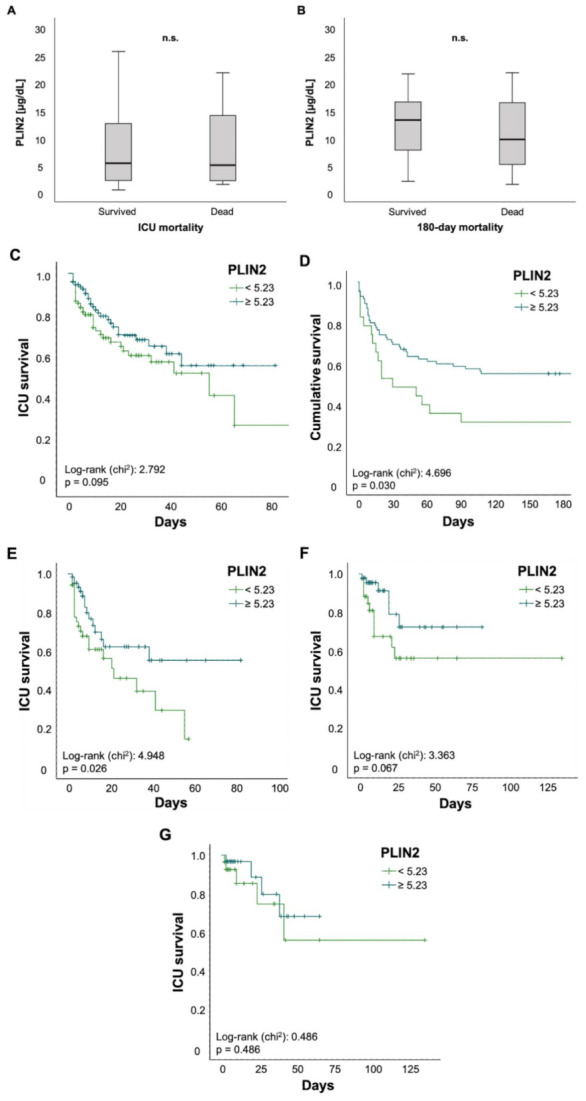
Serum concentrations of PLIN2 may predict ICU mortality in patients older than 65 years. (**A**–**G**) Neither total ICU- nor 180-day mortality was associated with significant changes in PLIN2 levels (**A**,**B**). Kaplan–Meier curve analysis showed a trend towards significancy for ICU mortality (**C**), whereas 180 day mortality was significant (**D**). Subgroup analysis revealed that reduced PLIN2 levels in patients older than 65 years may predict ICU mortality (**E**), whereas diabetic patients showed a trend towards significancy (**F**). PLIN2 was not able to predict mortality in patients with a body mass index above 30 (**G**).

Finally, we investigated the value of PLIN2 as a biomarker for predicting mortality in critically ill patients. There was no difference in PLIN2 serum levels among ICU patients who survived or those who died during their ICU stay (5.36 (0.48–59.5) µg/dL vs. 5.01 (1.49–48.3) µg/dL, *p* = 0.674; Figure 4A). The same applied to ICU patients who were alive 180 days after ICU admission or their counterparts who died within 180 days after initial ICU admission (13.21 (2.05–59.5) µg/dL vs. 9.66 (1.49–21.71) µg/dL, *p* = 0.117; Figure 4B). To further evaluate the predictive capability of PLIN2, we performed Kaplan–Meier curve analyses. These revealed that PLIN2 serum levels below the median PLIN2 concentrations of all patients (5.23 µg/dL) tended to indicate poorer ICU survival (log-rank *p* = 0.095; Figure 4C) and were displaying higher 180 day mortality rates (log-rank *p* = 0.03; Figure 4D). Encouraged by data highlighting the role of PLIN2 in the pathophysiology of age-related and metabolic diseases [6], we performed subgroup survival time analyses. Patients > 65 years with PLIN2 levels below median showed impaired ICU survival when compared to patients above the cut-off (log-rank *p* = 0.026**;** Figure 4E). Moreover, Kaplan–Meier curve analysis demonstrated a trend towards impaired ICU survival in diabetic patients with lower PLIN2 serum levels (log-rank *p* = 0.067; Figure 4F), whereas no such effect was seen in the subgroup of non-diabetics (data not shown) or patients with a body mass index ≥30 kg/m^2^ (log-rank *p* = 0.486; Figure 4G). Because serum PLIN2 was strongly negatively correlated with serum lipase and the presence of acute pancreatitis, we investigated whether in the subgroup of patients with acute pancreatitis serum PLIN2 was associated with increased ICU mortality. However, Kaplan–Meier curve analysis did not show a difference between pancreatitis patients with serum PLIN2 levels below or above the median concentration (log-rank *p* = 0.617; Figure S4A). Because serum PLIN2 was diminished in patients with severe respiratory failure, defined as PaO_2_/FiO_2_ < 100, we analyzed if among this subgroup serum PLIN2 concentrations below or above the median were associated with ICU mortality. However, Kaplan–Meier curves analysis did not show a significant difference (log-rank *p* = 0.206; Figure S4B).

## 4. Discussion

This is, to the best of our knowledge, the first study assessing serum PLIN2 in a representative cohort of critical illness at admission to a medical ICU. This study showed that PLIN2 is elevated in critically ill patients compared to controls. Most importantly, PLIN2 is capable of both independently indicating multiple organ dysfunction (MOD), defined as an increased SOFA score > 9 points, at ICU admission, and independently predicting MOD 48 h after PLIN2 measurement.

To evaluate whether PLIN2 may serve as a biomarker for critically ill patients we performed comprehensive analyses to rule out confounding effects. In terms of patient characteristics, we evaluated the interdependency of PLIN2 with (i) sex, (ii) age, (iii) BMI, (iv) dyslipidemia, and (v) insulin resistance and diabetes mellitus. Taken together, these sensitivity analyses indicate that serum PLIN2 concentrations are independent of various patient characteristics and comorbidities, indicating this biomarker is independent of relevant confounding factors. At the same time, serum PLIN2 does not appear to reflect metabolic dysregulation in critically ill patients.

(i) Because of growing evidence suggesting sex differences in the pathophysiology of critical illness [49,50,51], we investigated whether PLIN2 concentrations show a different distribution based on sex. In contrast to recent data stating that PLIN2 levels were higher in women, especially in patients below 79 years [32], we did not detect any difference of serum PLIN2 in both sexes.
(ii) Previous work speculated on age-dependent changes of PLIN2 expression and linkage to sarcopenia [33,34]. To assess that, we analyzed serum PLIN2 concentrations in various age groups and did not detect significant differences.
(iii) As BMI and mortality act in a “J-shaped” dependence and, because overweight and moderate obesity appear to be protective factors in critically ill patients, called the “obesity paradox” [36,52,53], we correlated serum PLIN2 with BMI and analyzed PLIN2 in different BMI subgroups (underweight vs. normal vs. overweight vs. obese; data not shown). Serum PLIN2 did not show any significant differences in these analyses, which is in contrast to previously published data [32].
(iv) PLIN2 acts as a regulator in lipid metabolism [5,7,15,16] and previous murine and human studies have shown that PLIN2 dysregulation can be associated with lipid storage malfunction diseases [7,14,17,18,19,20,21,22]. Surprisingly and despite PLIN’s function as a lipid droplet protein, we did not detect an association of serum PLIN2 with classical markers of lipid metabolism such as cholesterol, LDL or triglycerides. These results are not in line with experimental studies, which showed reduction of hepatic steatosis or lower triglycerides in serum and liver after knock-out or downregulation of PLIN2 expression [6,7,9,19,54].
(v) Insulin resistance and diabetes mellitus are important modulators of mortality and morbidity in critically ill patients [36]. Experimental research suggests that PLIN2 is involved in the pathophysiology of insulin resistance [12,13,14]. This encouraged us to analyze the relationship between pre-existent diabetes and PLIN2 serum levels. Of note, serum PLIN2 concentrations were independent of the presence of diabetes. Moreover, we did not observe a correlation between PLIN2 levels and HbA1c levels, but a non-significant trend towards a rather weak correlation with HOMA-IR. Interestingly, a recently published study reports similar results, with no observed association with DM, but a significant correlation between PLIN2 and HOMA-IR [32]. Another group described higher PLIN2 levels in diabetic patients with NAFLD compared to patients without NAFLD, and correlations with age, waist circumference, triglycerides and HOMA-IR. However, these results are difficult to compare to our study because we did not assess our patients for the presence of NAFLD.

PLIN2 concentrations were not only independent of the above-mentioned patient characteristics, but also independent of various established inflammatory markers (e.g., CRP and PCT). Of note, the missing association of PLIN2 with CRP was recently confirmed in an Italian cohort [32]. Additionally, PLIN2 levels were not influenced by the infectious source (Figure S2). Collectively, our multivariable analyses (Table 3) suggest a promising potential of PLIN2 as a biomarker for multiple organ dysfunction, irrespective of relevant confounders and commonly used biomarkers. Most excitingly, the predictive potential of MOD after 48 h could aid the intensivist in risk stratification of ICU patients at the time of PLIN2 measurement (Table 3).

To further understand the applicability of PLIN2, we performed a comprehensive analysis of various components of ICU parameters and organ dysfunction. Most components of the SOFA score, i.e., defining cardiovascular, hepatic, renal and coagulation failure, did not show consistent correlations with serum PLIN2. By comparison, we were surprised about the finding that patients with a Horovitz quotient ≤100 presented with decreased PLIN2 levels. We suspect that during a state of severe critical illness and subsequent intense ventilation and medication support, compared to mild or moderate critical illness, PLIN2 expression could be downregulated due to multiple reasons that are difficult to dissect in such a setting. Moreover, this may also be a phenomenon of serious dysregulation of metabolism in moribund patients. Taken together, we were not able to define a relevant driving force associated with the observed changes in PLIN2 serum concentrations. Therefore, we next investigated whether changes in serum PLIN2 were associated with the presence of various comorbidities: (a) cardiovascular disease, (b) hepatic disease, (c) pancreatic disease, and (d) malignant disease.

(a) There has been evidence of PLIN2 involvement in the development of age-related vascular disease, such as atherosclerosis [6,15,23,24,25,26,27]. Recent studies highlighted the importance of PLIN2 in cardiomyocyte lipid accumulation [28] and were able to connect PLIN2 to coronary microvascular obstruction and infarct size in patients with ST-elevation myocardial infarction and major adverse cardiovascular events during follow-up [55]. Intrigued by these findings, we investigated whether arterial hypertension or coronary artery disease are associated with altered PLIN2 serum levels. However, our results did not prove any obvious association between vascular diseases and PLIN2 levels.
(b) Prompted by experimental research connecting changes in PLIN2 serum levels with hepatic diseases and alcohol consumption [20,21,54,56], we analyzed serum PLIN2 concentrations in ICU patients suffering from liver cirrhosis and patients with a history of alcohol abuse. However, we did not observe any connections between cirrhosis or alcohol abuse and PLIN2 levels. However, this may be due to missing statistical power because only 3.1% of our cohort had cirrhosis. Additionally, the majority of the mentioned studies assessed PLIN2 in NAFLD or non-alcoholic steatohepatitis (NASH) instead of cirrhosis, further reducing the comparability of the results.
(c) PLIN2′s activation state is regulated by pancreatic hormones. While catecholamines permit lipolysis via phosphorylation and dissociation of PLIN2, insulin inhibits lipolysis via dephosphorylation of PLIN2, hindering hormone-sensitive lipases in accessing the lipid droplets [21]. Of note, we observed a strong negative correlation between PLIN2 and lipase. Correspondingly, patients admitted due to acute pancreatitis presented with decreased PLIN2 concentrations. However, serum PLIN2 was not able to discriminate a mortality difference in the small subgroup of patients with acute pancreatitis (*n* = 13; Figure S4A). To the best of our knowledge, associations with pancreatic markers or disease have not been previously described. Taking physiological mechanisms into consideration, the inverse association of PLIN2 with lipase raises the question of whether this is due to pancreatitis and its associated multiple organ dysfunction or rather an effect of higher PLIN2 metabolization of serum lipases. Importantly, serum PLIN2 levels were not associated with norepinephrine demand. Moreover, the logistic regression analyses of PLIN2 and SOFA score > 9 points remained significant after adjustment for the norepinephrine demand (Table 3).
(d) Previous studies have demonstrated the role of PLIN2 as a tumor marker in different body fluids or in tumor tissue [6,57,58] for several malignant diseases, such as renal cell carcinoma [57,59,60,61], colorectal carcinoma [29] or lung adenocarcinoma [30]. In our ICU cohort, PLIN2 serum concentrations were elevated in patients with preexistent malignant disease. These consistent associations of serum PLIN2 with malignancy even during critical illness underline its potential capacity as a tumor marker for routine diagnostics.

Muscle weakness in ventilated patients with respiratory failure has been associated with poor outcome [62]. Different mechanisms leading to muscle weakness are, among others, infiltration of skeletal muscle tissue with adipose tissue (myosteatosis) or age-related muscle atrophy (sarcopenia), although both factors should be understood as complementary and interacting [62,63,64]. However, assessing muscle weakness may be challenging in the ICU setting. Using whole-body dual-energy X-ray absorptiometry (DXA) scans, a recent study observed associations between PLIN2 and fat mass parameters. While they did not observe an association between PLIN2 and visceral adipose tissue, there was a strong correlation between PLIN2 and subcutaneous adipose tissue. However, body composition analyses via DXA-scans are prone to both over- and underestimation, especially in non-healthy individuals or markedly obese women [65,66].

Computed tomography (CT) scan body composition analyses emerge as an attractive and potentially more reliable alternative in the ICU setting [41,42,64,67,68,69]. Subsequently, a well-characterized subset of our ICU cohort underwent CT-scan body composition analyses. Taken together, we observed no clinically meaningful associations in this rather small subgroup (*n* = 36). Although this is contrary to previous data [6,32], the missing association of PLIN2 with body composition markers is consistent with the missing association with markers of metabolic dysfunction. Of note, previous studies assessed *Plin2* expression in skeletal muscle whereas, to the best of our knowledge, PLIN2 was not assessed as a serum biomarker in the context of muscle weakness or sarcopenia yet [31,33,34]. Although our hypothesis that PLIN2 associates with sarcopenia was not proven, this independency might further strengthen PLIN2′s role as a potential biomarker for multiple organ dysfunction.

With ICU mortality being the most substantial endpoint, we did not observe significant differences in serum PLIN2 levels in patients surviving versus deceasing on the ICU. Additionally, analyses of 180 days post-discharge from the ICU did also not reveal significant changes. However, Kaplan–Meier curve analyses revealed that PLIN2 may have predictive value for ICU mortality in patients > 65 years. Although this is consistent with studies previously reporting about the relevance of PLIN2 in age-related diseases [6], those patients with serum PLIN2 levels above the median had worse outcomes, which may reflect the detrimental outcome of patients with severe respiratory failure. Although we discovered significant survival differences for PLIN2 and 180 day mortality, this finding should be interpreted with caution. Survival post discharge at 180 days is prone to multifactorial confounders which cannot be accounted for in this type of study. Moreover, those patients with serum PLIN2 levels above the median had a better survival, again, reflecting the role of severe respiratory failure that was associated with reduced serum PLIN2. Collectively, PLIN2 does not appear to be a useful mortality marker.

To further evaluate the diagnostic role of serum PLIN2 in critically ill patients, we compared its performance with other experimental biomarkers that were previously described in our ICU cohort. Such cross-validation might help to further characterize the usefulness of PLIN2 as an adjunct to currently used biomarkers as the latter still lack in predictive power, specificity and sensitivity when it comes to differentiating the various etiologies of critical illness [70,71]. First, we observed a correlation between PLIN2 and Myostatin, a protein negatively regulating skeletal muscle growth, which was reduced in ICU patients and mechanically ventilated patients, and was also an independent prognostic marker for overall survival [47]. Interestingly, this is consistent with the finding that decreased PLIN2 levels in critically ill patients were associated with severe oxygenation failure. Second, we observed a negative correlation between PLIN2 and Adiponectin, an adipokine [45,46]. This is coherent with previous literature, describing decreased Adiponectin levels in critically ill patients [72,73,74,75]. Moreover, Adiponectin is an independent positive predictor for short-term and overall survival, although the current literature is still ambiguous and controversial as to whether decreased or increased Adiponectin levels are predictors of sepsis and outcome [45,46,72,75]. Third, we observed an association between PLIN2 and Symmetric and Asymmetric dimethylarginine (SDMA/ADMA), which were previously described reflecting the vascular tone and endothelial dysfunction, being elevated in critically ill patients, and predicting short-term and long-term survival [76,77]. Interestingly, ADMA has additionally been reported in various contexts, such as chronic kidney disease [78], cardiovascular disease (e.g., hypertension, atherosclerosis and coronary artery disease) and diabetes mellitus [79,80]. Future studies are warranted to assess whether PLIN2 measurements can enhance the diagnostic and prognostic value of ADMA, especially in the context of the observed changes in PLIN2 levels in severe respiratory failure.

An important limitation is the cross-sectional nature of our study design. Although our cohort is well characterized with adequate statistical power for multiple clinical parameters and endpoints, and offering clinical and laboratory data from two time points (ICU admission and ICU day 3), our cohort cannot replicate the complete ICU course (e.g., data from farther time points) which, due to its nature, is rather complex. Another weakness of our study, given its exploratory approach, is that the data were gathered from a single ICU, which does not consider external influencing factors and does not allow for generalizability. However, our study population includes heterogeneous disease etiologies over a recruitment period of five years, further increasing the representative capacity of our cohort. Although this may conceal disease-specific confounders, at the same time it also provides generalizability of the results. Another limitation is, due to the retrospective nature of our study, that our cohort stems from the previous era of sepsis definition. To reduce this limitation, we retrospectively applied the current Sepsis-3 criteria whereas, however, the treatment was conducted according to current guidelines at that time. A further major weakness is the small sample size of our control group. However, our focus was the comprehensive evaluation in our well-characterized cohort of critically ill patients. Another restriction is that the observed association of serum PLIN2 with sepsis was rather weak and does not appear to be clinically meaningful. Moreover, the small but statistically significant difference of serum PLIN2 concentrations between septic and non-septic ICU patients is also clinically not meaningful and may be within the measurement error range of the used ELISA assay. Furthermore, elevated PLIN2 levels were consistently associated with MOD whereas decreased PLIN2 levels were associated with increased mortality in subgroups. This contradiction is partly explained by the marked decrease of PLIN2 concentrations in severe respiratory failure, which in turn results in increased mortality. However, critically ill patients have multiple dysfunctions of various systems (e.g., MOD, dysregulated metabolism, dysregulated hormonal balance and immune system dysfunction) that are interconnected with each other and at the same time poorly understood at a mechanistic level. Moreover, this dyshomeostasis is further affected by use of multiple medications and assist devices (e.g., respirator and renal replacement). In addition, due to the nature of a biomarker study, the underlying mechanisms of the observed associations remain unknown. Finally, validation of our findings in an independent cohort and different disease settings may be warranted to also investigate an optimal PLIN2 cut-off value.

## 5. Conclusions

Serum PLIN2 may be a useful marker of MOD both at ICU admission and after 48 h with potential for clinical risk stratification. Although these independent and predictive associations are intriguing, the pathomechanisms leading to the observed changes remain to be elucidated.

## Figures and Tables

**Table 1:** Correlations of clinical and laboratory parameters with PLIN2 serum concentrations at ICU admission.

Parameters	r	*p*
Demographics		
Age	0.064	0.305
Body mass index	−0.099	0.129
FBC and markers of inflammation		
MCHC	0.166	0.007 *
Platelets	−0.135	0.030 *
WBC	−0.109	0.080
C-reactive protein	0.025	0.688
Procalcitonin	−0.023	0.751
Interleukin 6	0.050	0.486
Interleukin 10	0.092	0.303
TNF-α	−0.165	0.157
Electrolytes and renal system		
Sodium	−0.101	0.105
Potassium	−0.143	0.021 *
Urea	0.013	0.839
Creatinine	−0.042	0.499
HPB system		
Albumin	−0.020	0.805
INR	0.004	0.955
Bilirubin, total	−0.056	0.369
γGT	−0.099	0.115
AST	−0.072	0.263
Lipase	−0.509	<0.001 *
Cardiopulmonary system		
NTproBNP	0.024	0.785
Norepinephrine demand at day 1 (µg/kg/min)	0.066	0.322
Horovitz quotient (PaO_2_/FiO_2_)	0.202	0.052
Ventilatory FiO_2_ demand	−0.224	0.026*
Metabolism and endocrinology		
Glucose	0.066	0.292
HbA1c	−0.090	0.355
Insulin	−0.035	0.720
C-Peptide	−0.095	0.330
HOMA IR	−0.173	0.077
Cholesterol	−0.011	0.868
HDL-cholesterol	0.062	0.533
LDL-cholesterol	0.101	0.309
Triglycerides	−0.094	0.170
ICU parameters		
Days on ICU	0.132	0.034 *
SOFA day 1	0.149	0.113
SOFA day 3	0.261	0.014 *
APACHE-II day 1	−0.102	0.151
APACHE-II day 3	0.240	0.020 *

Spearman rank correlation test was used to calculate significant correlations of positive and negative nature. *p*-values < 0.05 were considered statistically significant and were highlighted (“*”). Abbreviations: ICU: intensive care unit; FBC: Full blood count; MCHC: Mean corpuscular hemoglobin concentration; WBC: White blood cell count; TNF: Tumor necrosis factor; GFR: Glomerular filtration rate; HPB: Hepato-Pancreato-Biliary; INR: International normalized ratio; γGT: Gamma-glutamyl transpeptidase; AST: Aspartate aminotransferase; NTproBNP: N-terminal pro B-type natriuretic peptide; FiO_2_: Fraction of inspired oxygen; HbA1c: Glycosylated hemoglobin A1; HOMA: Homeostatic model assessment; HDL: high-density lipoprotein; LDL: low-density lipoprotein; SOFA: Sequential organ failure assessment; APACHE-II: acute physiology and chronic health evaluation II.

**Table 2:** Baseline patient characteristics at ICU admission divided by the presence of sepsis.

Parameters	All Patients *n* = 259	Non-Sepsis *n* = 93	Sepsis *n* = 166	*p*
Female (%)	40.5%	39.8%	41%	n.s.
Age (years)	63 (18–89)	60 (18–85)	64 (21–89)	n.s.
Body mass index (kg/m^2^)	26 (15.9–86.5)	25.8 (15.9–53.3)	26.1 (17.1–86.5)	n.s.
**Comorbidities**				
Arterial Hypertension (%)	23.9	26.9	22.3	n.s.
Diabetes mellitus (%)	31.3	32.3	30.7	n.s.
Coronary artery disease (%)	12.7	15.1	11.5	n.s.
COPD (%)	17.4	21.5	15.1	n.s.
Liver cirrhosis (%)	3.1	5.4	1.8	n.s.
Malignant disease (%)	11.2	7.5	13.3	n.s.
**Clinical parameters**				
Mechanical ventilation demand at day 1 (%)	72.2	64.5	76.5	0.039
Norepinephrine demand at day 1 (%)	59.1	46.2	66.3	<0.001
Norepinephrine demand at day 1 (µg/kg/min)	0 (0–2.4)	0 (0–2.4)	0.1 (0–1.5)	0.001
Renal replacement therapy demand at day 1 (%)	27.4	18.3	32.5	0.010
Renal replacement therapy (days)	0 (0–37)	0 (0–21)	0 (0–37)	0.006
APACHE-II score at day 1	17 (2–43)	14 (2–33)	19 (3–43)	<0.001
APACHE-II score at day 3	19 (0–36)	12 (0–28)	22 (6–36)	<0.001
SOFA score at day 1	9 (0–19)	7 (0–17)	10 (3–19)	<0.001
SOFA score at day 3	9 (0–18)	6 (0–15)	10 (1–18)	<0.001
Days on ICU	8 (2–137)	6 (2–45)	10 (2–137)	<0.001
Death on ICU (%)	24.7	17.2	28.9	0.036
180-day mortality (%)	20.8	17.2	22.9	n.s.
Observation period (days)	137 (1–884)	195.5 (1–883)	110 (1–884)	n.s.
Overall mortality (%)	47.5	34.0	54.8	0.002
**Laboratory data at day 1:**				
WBC [×10^3^/μL]	12.7 (0–149)	11.4 (1.8–29.6)	13.1 (0–149)	0.011
C-reactive protein [mg/dL]	97 (5–230)	17 (5–230)	161.5 (5–230)	<0.001
Procalcitonin [ng/mL]	0.8 (0–248)	0.2 (0–100)	2.7 (0.1–248)	<0.001
Creatinine [mg/dL]	1.4 (0.2–21.6)	1 (0.2–15)	1.6 (0.2–21.6)	0.025
Creatinine GFR [mL/min]	54 (2–60)	60 (6–60)	38 (2–60)	0.004
INR [units]	1.2 (0.9–6.7)	1.2 (0.9–6.7)	1.2 (0.9–4.6)	n.s.
Albumin [mg/dL]	27 (1.6–61.4)	30.1 (1.6–48.5)	25.6 (5–61.4)	0.005
Lactate [mmol/l]	1.6 (0.4–21.9)	1.8 (0.6–18.1)	1.5 (0.4–21.9)	0.094
PLIN2 [µg/dL]	5.23 (0.48–59.5)	4.86 (1.4–32.4)	5.47 (0.48–59.5)	0.021

For quantitative values, median and range (in parenthesis) are given. Abbreviations: COPD: chronic obstructive pulmonary disease; APACHE-II: acute physiology and chronic health evaluation II; SOFA: sequential organ failure assessment; ICU: intensive care unit; WBC: white blood cell count; GFR: glomerular filtration rate; INR: international normalized ratio; PLIN2: Perilipin 2.

**Table 3:** Logistic regression analyses for serum PLIN2 concentrations above the median concentration and clinically relevant outcome parameters.

**Sepsis occurrence at ICU admission**	**OR (95% CI)**	***p***
Unadjusted	1.72 (1.03–2.88)	0.038
Adjusted for age and DM	1.71 (1.02–2.87)	0.042
Adjusted for CRP	1.75 (0.94–3.28)	0.079
Adjusted for CRP and PCT	2.07 (0.98–4.38)	0.058
Adjusted for norepinephrine demand	1.73 (1.01–2.98)	0.047
**SOFA > 9 points at ICU admission**	**OR (95% CI)**	***p***
Unadjusted	3.13 (1.36–7.20)	0.007
Adjusted for age and DM	2.96 (1.27–6.88)	0.012
Adjusted for CRP	3.07 (1.33–7.09)	0.009
Adjusted for CRP and PCT	2.62 (0.67–10.24)	0.166
Adjusted for norepinephrine demand	2.72 (1.08–6.89)	0.035
**SOFA > 9 points at day 3**	**OR (95% CI)**	***p***
Unadjusted	3.08 (1.25–7.60)	0.015
Adjusted for age and DM	2.91 (1.16–7.29)	0.023
Adjusted for CRP	2.93 (1.17–7.32)	0.021
Adjusted for CRP and PCT	15.93 (2.85–88.93)	0.002
Adjusted for norepinephrine demand	2.79 (1.05–7.42)	0.040

Association between serum PLIN2 concentrations above the median of 5.23 µg/dL at the day of admission and sepsis occurrence at ICU admission (upper panel), SOFA score > 9 points at ICU admission (middle panel) and SOFA score > 9 points at ICU day 3 (i.e., 48 h after PLIN2 measurement; lower panel). The covariates (laboratory data and norepinephrine demand in µg/kg/min) are from the day of admission (same timepoint as PLIN2 measurement). Abbreviations: ICU: intensive care unit; OR: odds ratio; CI: confidence interval; DM: Diabetes mellitus; CRP: C-reactive protein; PCT: Procalcitonin; SOFA: sequential organ failure assessment.

**Table 4:** Correlations between PLIN2 serum levels and CT-scan body composition markers.

r|p	VAT [mm^2^]	SAT [mm^2^]	Skeletal Muscle [mm^2^]	Skeletal Muscle Mean HU	L3SMI
All patients	−0.004|0.983	−0.160|0.353	−0.152|0.377	−0.213|0.213	−0.095|0.592
BMI < 30 kg/m^2^	−0.015|0.942	−0.214|0.305	−0.002|0.991	−0.261|0.208	0.033|0.875
BMI ≥ 30 kg/m^2^	−0.750|0.020 *	−0.517|0.154	−0.567|0.112	0.259|0.500	−0.533|0.139
Age < 65 years	−0.030|0.898	−0.156|0.500	−0.138|0.552	−0.152|0.510	−0.072|0.770
Age ≥ 65 years	0.061|0.830	−0.036|0.899	−0.146|0.603	−0.152|0.589	−0.079|0.781

Spearman rank correlation test was used to calculate significant correlations of positive and negative nature. Abbreviations: CT: computed tomography; VAT: visceral adipose tissue; SAT: subcutaneous adipose tissue HU: Hounsfield unit; L3SMI: skeletal muscle index at third lumbar vertebra; BMI: body mass index. *p*-values < 0.05 were considered statistically significant and were highlighted (“*”).

## Data Availability

The data are available from the corresponding author on reasonable request.

## Supplementary Materials

The following are available online at https://www.mdpi.com/article/10.3390/biomedicines9091210/s1, Figure S1: Patients admitted to the ICU due to acute pancreatitis showed decreased PLIN2 serum levels compared to patients admitted due to other reasons, Figure S2: Serum PLIN2 levels at ICU admission in septic patients, Figure S3: PLIN2 serum levels and urine output, Figure S4: PLIN2 serum concentrations and ICU mortality, Table S1: Correlations of experimental biomarkers with PLIN2 serum concentrations at ICU admission, Table S2: Main cause for admission to the intensive care unit.
