# Supplementary material for: Serum Perilipin 2 (PLIN2) Predicts Multiple Organ Dysfunction in Critically Ill Patients
Source: Biomedicines. 2021 Sep 13;9(9):1210. doi: 10.3390/biomedicines9091210 (PMC8468514; doi:10.3390/biomedicines9091210)
Supplement: Supplementary file 1 [file biomedicines-09-01210-s001.zip › biomedicines-1321761-supplementary.pdf]

biomedicines

MDPI

Serum Perilipin 2 (PLIN2) Predicts Multiple Organ Dysfunction in Critically Ill Patients

Supplementary data:

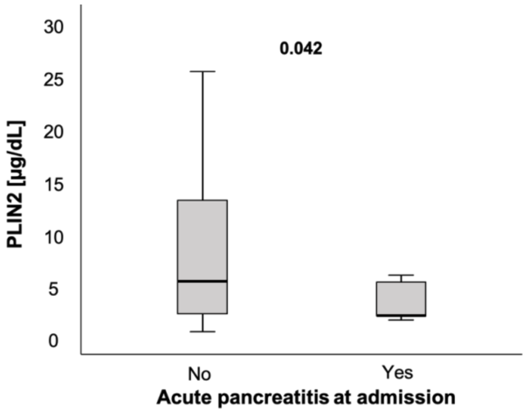

Figure S1. Patients admitted to the ICU due to acute pancreatitis showed decreased PLIN2 serum levels compared to patients admitted due to other reasons.

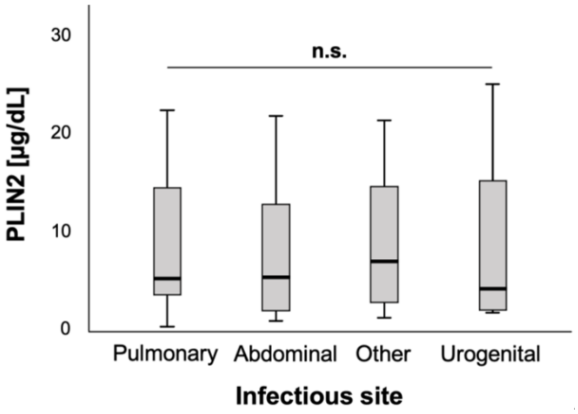

Figure S2. Serum PLIN2 levels at ICU admission in septic patients. PLIN2 serum concentrations were not different in patients with differing infectious sources.

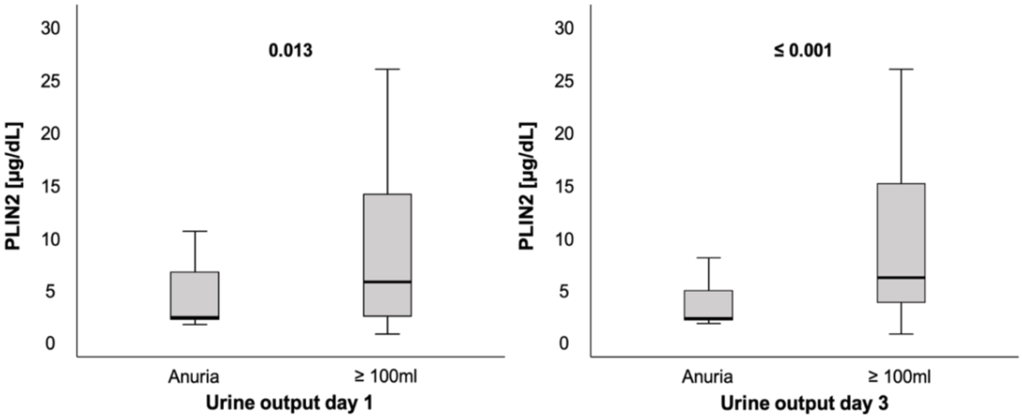

(A) (B)

Figures S3. PLIN2 serum levels and urine output. **(A)** PLIN2 serum concentrations at day 1 were decreased in patients that were anuric at day of ICU admission**. (B)** Patients that were anuric at day 3 showed even lower PLIN2 levels at ICU admission.

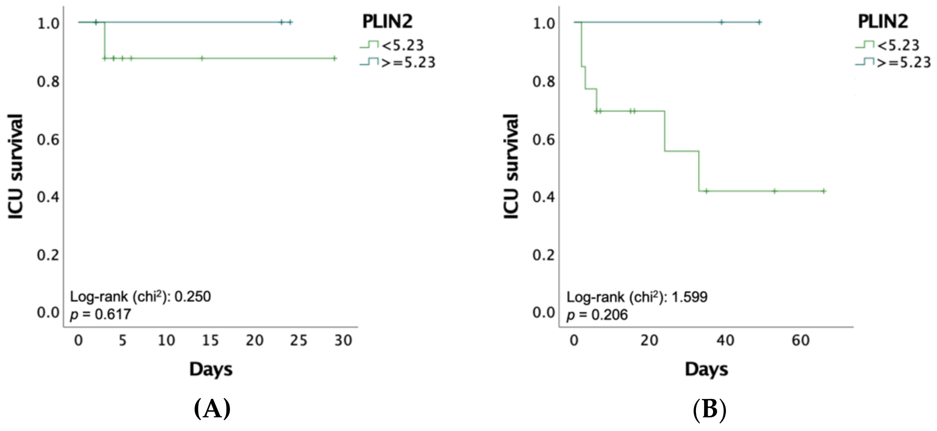

Figure S4. PLIN2 serum concentrations and ICU mortality. **(A)** Serum PLIN2 levels did not discriminate an ICU mortality difference in patients admitted due to acute pancreatitis. **(B)** Serum PLIN2 did not predict ICU mortality in patients with severe respiratory failure, defined as PaO_2/FiO_2 <100.

Biomarker	r	*p*
Adiponectin	-0.273	0.007*
RBP4	0.202	0.065
APRIL	0.135	0.056
sFRP5	-0.175	0.007*
Calprotectin	0.097	0.125
Myostatin	0.160	0.010*
ADMA	-0.197	0.002*
SDMA	-0.154	0.017*
suPAR	-0.049	0.499

Table S1. Correlations of experimental biomarkers with PLIN2 serum concentrations at ICU admission. Spearman rank correlation test was used. *Abbreviations: RBP4: Retinol binding protein 4; APRIL: A proliferation-inducing ligand; sFRP5: Secreted frizzled-related protein 5; ADMA: Asymmetric dimethylarginine; SDMA: Symmetric dimethylarginine; suPAR: Soluble urokinase-type plasminogen activator receptor.*

**Critical illness other than sepsis n (%)**	**93 (35.9%)**
**Etiology n (relative /absolute):**	
Cardiopulmonary disease	32 (34.4% / 12.4%)
Decompensated liver cirrhosis	16 (17.2% / 6.2%)
Acute pancreatitis	13 (14% / 5%)
Severe gastrointestinal bleeding	6 (6.5% / 2.3%)
Acute liver failure	4 (4.3% / 1.5%)
Other	22 (23.7% / 8.5%)
**Sepsis n (%)**	**166 (64.1%)**
**Infectious site n (relative / absolute):**	
Pulmonary	92 (55.4% / 35.5%)
Abdominal	32 (19.3% / 12.4%)
Urogenital	7 (4.2% / 2.7%)
Other	35 (21.1% / 13.5%)

Table S2. Main cause for admission to the intensive care unit.
